# Mitigating the Risk of Adverse Effects Related to Augmentation Therapy for Resistant Major Depressive Disorder: A Case Report

**DOI:** 10.3390/medicina58030438

**Published:** 2022-03-17

**Authors:** Collin J. Amundson, Robert Knight, Georgina M. Ybarra, Jacques Turgeon, Jennifer M. Bingham

**Affiliations:** 1 Ken R. Coit College of Pharmacy, University of Arizona, Tucson, AZ 85721, USA; camundson@pharmacy.arizona.edu (C.J.A.); jbingham@trhc.com (J.M.B.); 2 Tabula Rasa HealthCare, MedWiseRx, 100 N Stone Ave Suite 109-222, Tucson, AZ 85701, USA; rknight@trhc.com (R.K.); gmybarra@sinfoniarx.com (G.M.Y.); 3 Tabula Rasa HealthCare, Precision Pharmacotherapy Research & Development Institute, 13485 Veterans Way, Orlando, FL 32827, USA; 4 Tabula Rasa HealthCare, Office of Translational Research & Residency Programs, 228 Strawbridge Dr, Moorestown, NJ 08057, USA

**Keywords:** drug–drug interactions, antidepressants, depression, restless legs syndrome, antipsychotics

## Abstract

Polypharmacy of psychotropic medications predisposes older adults to adverse drug events (ADEs). One contributing factor is inhibition of metabolic pathways between substrates (competitive inhibition) or between substrates and inhibitors of the same cytochrome P450 (CYP450) isoforms. The purpose of this case report is to demonstrate observed sedation and difficulty concentrating from augmentation therapy for resistant major depressive disorder (MDD) and to highlight the value of clinical tools to identify opportunities for treatment optimization to reduce ADEs. The pharmacist identified significant medication burden and competitive inhibition of drug metabolism in the CYP450 system during a telehealth medication therapy management consultation with a 69-year-old male. The pharmacist recommended clinical monitoring and communicated concerns about medication-induced sedation, difficulty concentrating, and other medication-related problems (MRP) to providers. Several recommendations were implemented which helped improved patient’s outcomes. Individualizing MDD pharmacotherapy based on pharmacokinetic and pharmacodynamic drug interactions and geriatric dosage considerations may lead to better outcomes and tolerability among older adults.

## 1. Introduction

Polypharmacy, complicated by drug interactions, interferes with achieving therapeutic goals of psychiatric treatment among older adults [1,2,3]. Most antidepressants and antipsychotics used for major depressive disorder (MDD) potentially have pharmacokinetic drug interactions through the cytochrome P450 (CYP450) system, especially CYP3A4, CYP2D6, and CYP2C19 [4,5,6,7]. Collaboration with pharmacists has reduced adverse events via drug–drug interaction campaigns in other countries, and optimized medication use via medication therapy management (MTM) services where medication therapy regimens are reviewed and optimized with the patient [8,9]. Patients are eligible for MTM services if they have three or more chronic conditions, if they take eight or more maintenance medications or if they spend US $4696 or more on medications. In this case presentation, the pharmacist identified culpable medications through using an advanced clinical decision support system (MedWise^®^: Tabula Rasa HealthCare, Moorestown, NJ, USA) that enables them to rapidly assess the clinical significance of drug interactions impacting medication burden (i.e., the likelihood that the interaction will lead to adverse effects in the patient). Herein, we describe a patient experiencing adverse drug effects (ADEs) from resistant MDD, exacerbated by add-on pharmacotherapy, leading to multiple clinically important pharmacokinetic and pharmacodynamic drug interactions.

## 2. Case Presentation

### 2.1. Past Medical History

A 69-year-old male with a history of ischemic stroke, chronic obstructive pulmonary disease (COPD), mild cognitive impairment, major depressive disorder (MDD) of moderate severity, peripheral neuropathy (PN), gastroesophageal reflux disease (GERD), type 2 diabetes mellitus (T2DM), restless legs syndrome (RLS), stable ischemic heart disease (SIHD), and hypertension (HTN) was consulted by a telehealth pharmacist as part of a routine medication therapy management (MTM) service provided by their prescription drug benefit plan.

### 2.2. Differential Diagnosis and Treatment

During the initial consultation, the patient reported increased sedation, lightheadedness, and difficulty concentrating throughout the day over the past six months, which had affected his activities of daily living. The patient reported a 110/60 mmHg blood pressure. He denied angina, prolonged fatigue after exercise, orthostatic hypotension, or history of iron deficiency anemia. Prior to the pharmacist’s consultation, he was treated by a psychiatrist, neurologist, cardiologist, and primary care provider (PCP). A list of medications pre-pharmacist consultation is shown in Table 1, and the patient reported adherence to all medications. The patient had no evidence of having trialed any other antidepressant other than escitalopram or bupropion.

### 2.3. Initial Consultation

The pharmacist reconciled the medication list and assessed for ADEs, multi-drug interactions, drug–disease interactions, and medication safety-related problems using an advanced clinical decision support system (CDSS, MedWise^®^). The clinical decision support system allowed the pharmacist to simultaneously assess cytochrome P450 interactions, risks for drug-induced long QT syndrome, sedative burden, and anticholinergic burden for numerous medications. The CDSS analyzes a beneficiary’s medication regimen in the context of these risk factors and identifies and resolves medication-related problems (MRPs) that are most likely contributing to the risk of ADEs and other negative utilization outcomes [10]. The CDSS noted competitive inhibition that placed the patient at increased risk for sedation, anticholinergic effects, and QT prolongation.

First, the pharmacist assessed principal medication-related causes of fatigue and sedation. Suspected causes of both fatigue and lightheadedness were medication burden, history of stroke, and uncontrolled major depressive disorder. Common medical diagnoses such as positional vertigo, orthostasis, Meniere’s disease, hypoglycemia, were of low suspicion as primary causes of the patient’s lightheadedness. The pharmacist counseled the patient about the association between his medications (e.g., escitalopram, aripiprazole, pramipexole, metoprolol succinate, memantine, gabapentin) and sedation, lightheadedness, and difficulty concentrating.

Next, upon assessment for anticholinergic complications related to aripiprazole and bupropion, the patient confirmed fatigue and lightheadedness. The pharmacist counseled on fall precautions related to dizziness and on reporting any symptoms of confusion, urinary retention, blurry vision, or palpitations to his physician.

Then, the pharmacist identified escitalopram as a contributor to the increased risk of QT prolongation. Upon reconciling the medication list the patient reported self-discontinuation of escitalopram for five days. He denied withdrawal symptoms and reported signs of worsened depression including dysthymia and lack of motivation. The pharmacist assessed for withdrawal symptoms and counseled the patient on the importance of continuously taking the medication to avoid withdrawal. The patient was advised to immediately refill their antidepressant. The pharmacist contacted the triage nurse at the PCP immediately to request an escitalopram refill and to discuss the interaction between omeprazole and escitalopram. The patient was not taking more than escitalopram 20 mg a day; therefore, no recommendations were made to monitor the QT interval. However, as omeprazole may increase concentrations of escitalopram twofold via the CYP2C19 metabolic pathway, leading to risk for QT prolongation, a de-escalation to famotidine monotherapy was suggested to avoid the interaction.

Due to competitive inhibition at the CYP3A4 isoenzyme, the patient was at increased risk of experiencing additive effects of aripiprazole given the interaction with amlodipine. Instead, the pharmacist advised the patient to separate administration times of amlodipine and aripiprazole to mitigate this avoidable adverse drug event.

In addition, an interaction between omeprazole, escitalopram, and aripiprazole was identified by the pharmacist. The pharmacist advised de-escalation of the acid-reducing therapy to famotidine monotherapy to the PCP as an opportunity to avoid the drug–drug interaction. The pharmacist also advised the provider to re-evaluate use of aripiprazole due to side effects, increased risk of supratherapeutic effects, and its black box warning related to the use of atypical antipsychotics in post-stroke patients. As iron deficiency can contribute to RLS, the pharmacist recommended monitoring of ferritin levels.

### 2.4. Follow-Up Consultation

During a follow-up telehealth consultation 2 months later, the pharmacist reconciled the medication list. The provider had accepted the pharmacist’s recommendations to monitor accordingly, making the following regimen changes over the course of several weeks.

1. Restarted escitalopram therapy.
2. Separated dose administration times of amlodipine and aripiprazole.
3. De-escalated the acid-reducing therapy to famotidine monotherapy.
4. Initiated ongoing assessment for aripiprazole and safer alternatives.

## 3. Discussion

### 3.1. Optimization of Resistant Major Depressive Disorder and Restless Legs Syndrome

Many older adults have under-treated MDD, which is often complicated by polypharmacy [11,12,13]. Complicated medication regimens can render anti-depressive therapy ineffective in the presence of polypharmacy [14]. While augmentation of antidepressants may have a treatment effect in older adults, lithium is the single agent that has supported efficacy in augmenting antidepressant therapy [15]. In the present case, the patient takes escitalopram as mainstay depression therapy, but is augmented by aripiprazole, memantine, and bupropion; perhaps by polypharmacy from multiple providers.

According to a recent review by Voineskos, Daskalakis, and Bumberger, selective serotonin reuptake inhibitors (SSRIs) or serotonin norepinephrine reuptake inhibitors (SNRIs) remain first line in treatment of resistant depression [3]. Use of memantine for depression with mild cognitive impairment but not dementia is supported by a single trial among 95 older adults with MDD [16]. Systematic review of the evidence, however, has not supported a treatment effect [17]. Given the side effect profile of memantine, use in the present case would be inappropriate.

Voineskos et al. cite the increasing use of second-generation antipsychotics for augmentation of resistant major depression therapy [3]. Quetiapine is approved by the U.S. Food and Drug Administration for this indication. Two underpowered trials funded by the manufacturer of aripiprazole suggest only a small benefit of aripiprazole 2–20 mg per day in improving reported symptoms of depression [18,19]. Results have not been replicated in a large randomized controlled trial to date. Alternatively, bupropion has been proposed for addition to SSRIs or SNRIs to boost treatment response [20]. A randomized clinical trial by Clayton and colleagues examined aripiprazole when combined with SSRI/SNRI or with bupropion and found over 90% of patients experienced at least one adverse event with the most common being fatigue [21]. It is unclear whether our patient was benefitting from the combination of memantine, bupropion, and aripiprazole with escitalopram therapy for depression; however, he was absolutely experiencing adverse effects interfering with activities of daily living associated with concomitant use of all three medications. Initiating augmentation therapy for depression necessitates careful assessment and follow-up of clinical utility. Of note, second generation antipsychotics carry a black box warning for use in some older adults for risk of increased mortality from stroke.

Diagnosis of restless legs syndrome complicates treatment of major depressive disorder. According to an updated clinical algorithm written by authors of the RLS guideline, antidepressants, antipsychotics, prokinetic agents, and first-generation antihistamines are common primary causes of restless legs syndrome [22]. Ruling out and correcting iron deficiency is first line; our patient had no history of iron deficiency. In the present case, the patient had been prescribed multiple antidepressants (escitalopram and bupropion) and a new antipsychotic (aripiprazole) for augmentation of major depressive disorder by different prescribers, creating the possibility of worsening RLS symptoms. Silber et al. also describe the augmentation effects or worsening of RLS symptoms emerging from long term use of non-ergot dopamine agonists for RLS [22]. Our patient had also been taking pramipexole for several years, which may lead to a rebound effect of RLS symptoms. Pharmacokinetic interactions increasing the concentrations of these neuroleptics may have predisposed the patient to poorly managed restless legs syndrome.

### 3.2. Pharmacodynamic Drug Interactions

Competing drug receptor targets may also contribute to our patient’s poor outcomes. While increasing serotonin in the synaptic cleft via the use of SSRIs combats depression and anxiety, our patient had been prescribed aripiprazole, which is known to partially agonize or fully antagonize the 5-HT_1A_, 5-HT_2A_, and 5-HT_2C_ serotonin receptors [23]. Bupropion, a mainstay of antidepressant augmentation, increases dopamine and norepinephrine via inhibition of reuptake, however, aripiprazole occupies the dopamine 2 receptor for weeks at a time, blocking the effect [24,25]. Our patient is also taking metoprolol. As a highly lipophilic beta blocker, metoprolol can easily access the central nervous system to cause blockade of norepinephrine, leading to sleep, concentration, memory, anxiety, and sedative effects; thus, it blocks the benefits of taking bupropion [26]. The patient noted taking a high dose of gabapentin, which inhibits calcium channels in the brain, leading to a depressant effect on all neurotransmission [27].

While our patient was started on pramipexole, a potent dopamine receptor agonist, he continued aripiprazole which occupies the D2 and D3 dopamine receptors thus potentially limiting pramipexole’s efficacy. Use of antidepressants can worsen RLS through the effects of augmentation, and recent guidance recommends limiting dopamine antagonism due to the rebound effect of stimulating dopamine receptors on RLS symptoms [22].

### 3.3. Pharmacokinetic Drug Interactions

Amlodipine and aripiprazole are metabolized by the same cytochrome P450 isoenzyme: CYP3A4. The stronger substrate (amlodipine) demonstrates greater affinity for the CYP3A4 isoenzyme. As a result, we expected to observe greater concentrations of the weaker substrate (aripiprazole) (see Table 1).

Another medication that increases concentrations of aripiprazole is bupropion. Aripiprazole and bupropion are both metabolized by CYP2D6 with the same level of affinity; however, when two drugs have the same affinity for an enzyme, the drug with the higher dose will out-compete and lower the metabolism of the lower strength drug [28].

Due to competitive inhibition by amlodipine at CYP3A4 and bupropion at CYP2D6, concentrations of aripiprazole are expected to be elevated in our patient. These increased aripiprazole concentrations may lead to undesired sedative effects and dopamine D2 receptor occupancy effects; thus, the pharmacist recommended re-evaluation of the use of this agent for major depressive disorder.

Furthermore, due to the stronger affinity of aripiprazole and bupropion to the CYP2D6 enzyme, metoprolol, a weaker CYP2D6 substrate is expected to have greater concentrations [29]. Aripiprazole has multiple drug receptor effects, including antagonism of the alpha-adrenergic receptors, which can lead to orthostasis when combined with agents with antihypertensive properties [30]. High affinity of aripiprazole and bupropion to the CYP2D6 isoenzyme predisposes our patient to higher concentrations of the lipophilic metoprolol, which can contribute to our patient’s CNS symptoms of lightheadedness, difficulty concentrating, and depression.

The pharmacist recommended close monitoring of blood pressure, pulse, and orthostatic hypotension to avoid hypotension from the metoprolol. They also recommended re-evaluation of aripiprazole as it contributes to increased concentrations of metoprolol.

Escitalopram, aripiprazole, bupropion, rosuvastatin, and omeprazole have all been associated with development of long QT syndrome [31,32,33]. The pharmacist advised de-escalation of omeprazole for acid-reducing therapy if clinically appropriate and re-assessment for simplification of this patient’s MDD medication regimen.

Lastly, a mechanism-based inhibitor (omeprazole) decreased the activity of the CYP2C19 isoenzyme involved in the metabolism of the substrate escitalopram. As a result, we should expect to observe greater concentrations of escitalopram, the parent drug. However, since escitalopram is normally transformed by the CYP2C19 isoenzyme into an active metabolite, under conditions of inhibition, lower concentrations of the active metabolite will be observed while concentrations of escitalopram will be higher [34]. Inhibition of metabolism by omeprazole may lead to an accumulation of escitalopram in the body and slowed clearance, predisposing our patient to side effects from escitalopram. The pharmacist recommended monitoring of the therapeutic effect of escitalopram, as conversion to the active metabolite is limited.

## 4. Conclusions

Polypharmacy of psychiatric medications predisposes patients to adverse drug event risk through receptor competition, to drug–drug and drug–gene interaction risk, and to combined adverse effect profiles. A clinical decision support system that aggregates medication burden facilitated analysis of the patient’s medication profile. One limitation to these results is that progress could not be monitored using scales such as the PHQ-9 or HAMD-17 as the consultation occurred outside of direct consultation with the psychiatrist. Considering all the potential pharmacokinetic and pharmacodynamic drug interactions, along with geriatric dosing considerations before starting new psychopharmacotherapy for older adults, may lead to improved outcomes via individualization.

## Figures and Tables

**Table 1:** Medication list and summary of affinity and CYP450 metabolic pathways pre-pharmacist consultation.

Drug	Dose	Frequency	Indication	CYP2B6	CYP 2C9	CYP2C19	CYP2D6	CYP3A4
Albuterol MDI *	1 puff (90 mcg)	q6h prn	COPD	(No expected CYTOCHROME P450 interactions due to route of administration)				
Fluticasone/umeclidinium/vilanterol *	1 puff (100/62.5/25 mcg)	Daily	COPD	(No expected CYTOCHROME P450 interactions due to route of administration)				
Famotidine	40 mg	Daily	GERD	(NON-CYTOCHROME P450)				
Omeprazole	20 mg	Daily	GERD			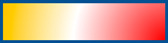		
Amlodipine	2.5 mg	Daily	HTN					
Lisinopril	5 mg	Daily	HTN	(NON-CYTOCHROME P450)				
Metoprolol	50 mg	Daily	HTN					
Aripiprazole	10 mg	Daily	MDD					
Bupropion SR	150 mg	BID	MDD					
Escitalopram	20 mg	Daily	MDD					
Memantine	5 mg	BID	Mild Cogn. Impair. *	(NON-CYTOCHROME P450)				
Gabapentin	800 mg	TID	Neuropathy	(NON-CYTOCHROME P450)				
Pramipexole	125 mcg	qHS	RLS	(NON-CYTOCHROME P450)				
Aspirin	81 mg	Daily	SIHD	(NON-CYTOCHROME P450)				
Isosorbide mononitrate ER	30 mg	Daily	SIHD	(NON-CYTOCHROME P450)				
Nitroglycerin	0.4 mg	prn angina	SIHD	(NON-CYTOCHROME P450)				
Rosuvastatin	40 mg	Daily	SIHD					
Canagliflozin/metformin XR	150 mg/ 1000 mg	Daily	T2DM	(NON-CYTOCHROME P450)				
				Affinity strength				
				Non-Competitive inhibition	Strong		Moderate	Weak

Key: mg = milligram; MDI = metered dose inhaler; mcg = microgram; XR = extended-release; SR = sustained-release; ER = extended-release; PRN = as needed; BID = twice daily; TID = three times a day; qHS = every evening at bedtime; CYP = cytochrome P450; COPD = chronic obstructive pulmonary disease; GERD = gastroesophageal reflux disease; HTN = hypertension; MDD = major depressive disorder; RLS = restless legs syndrome; SIHD = stable ischemic heart disease; T2DM = type 2 diabetes mellitus; * mild cognitive impairment.
